# Evaluation of Risk Factors for Dupilumab-Associated Ocular Sequelae in the Treatment of Atopic Dermatitis

**DOI:** 10.7759/cureus.76132

**Published:** 2024-12-21

**Authors:** Varsha Reddy, Alexa Lum, Katerina Kitsios, Swarna Shil, Alanna Nattis

**Affiliations:** 1 College of Osteopathic Medicine, Michigan State University, East Lansing, USA; 2 Ophthalmology, SightMD, Babylon, USA; 3 Ophthalmology, Good Samaritan Hospital Medical Center, Islip, USA

**Keywords:** atopic dermatitis, dupilumab, dupilumab adverse reaction, managing atopic dermatitis, ocular sequelae

## Abstract

Dupilumab is a monoclonal antibody that inhibits interleukin-4 (IL-4) and interleukin-13 (IL-13) signaling and is used in the treatment of moderate-to-severe atopic dermatitis (AD) in those six months or older who are uncontrolled on or cannot tolerate topical treatments. Ocular surface disease is a recognized adverse effect of dupilumab, yet few studies describe the risk factors for developing ocular adverse effects. There are no standardized recommendations for monitoring patients on this medication. This study aims to highlight the risk factors associated with the development of dupilumab-associated ocular surface disease (DAOSD) described in the literature. The PubMed and ScienceDirect databases were searched in April 2024 using key search terms. Nine articles were included after deduplication, title/abstract screening, full-text review, and quality appraisal. Studies were included if they were written in English and discussed risk factors for the ocular side effects of dupilumab. Studies were excluded if they discussed other biological agents or ocular conditions of other origins. Out of the nine studies analyzed, six described prior history of ocular disease as a risk factor for developing DAOSD. Severe AD was highlighted as a risk factor in five out of nine studies. Elevated total immunoglobulin E (IgE) levels and eosinophil count were described as risk factors in four out of nine studies. Three studies cited facial or eyelid eczema, and two studies highlighted family history of atopy as having an association with the development of DAOSD. One study described high levels of chemokines, as well as the personal history of other atopic conditions, as independent risk factors. While the etiology of DAOSD is not fully understood, past studies have elucidated potential risk factors for its development. Those being treated with dupilumab for AD have higher severity or refractory disease, and the discontinuation of treatment due to ocular side effects may have implications on the quality of life for these patients. Additional studies are needed to better understand the risk factors for DAOSD and prevent further complications.

## Introduction and background

Atopic dermatitis (AD) is the most common cause of skin disorders worldwide and affects patients of all ages and backgrounds [1]. This multifactorial disease involves dysfunction of the epidermal barrier, T2 helper (Th2) cell activation, and, often, genetic susceptibility. Patients often experience multiple coexisting conditions, which include, but are not limited to, allergic rhinitis, asthma, and food allergies [1]. Although a cure is not yet available, several new and innovative therapies have been developed to tackle its complex pathophysiology, including dupilumab.   

Dupilumab is a human monoclonal antibody that inhibits interleukin-4 (IL-4) and interleukin-13 (IL-13) signaling through receptor antagonism of a mutual IL-4ɑ subunit. IL-13 is considered an effector cytokine that induces disease, whereas IL-4 promotes the growth of the CD4+ Th2 cell population [2]. The subsequent potentiation of the Th2 response is strongly implicated in the development of AD, and dupilumab’s ability to suppress the IL-4/IL-13 signaling pathway makes it a suitable treatment option for this complex skin disorder [2]. In addition to attenuating these biomarkers, dupilumab has been shown to reduce T cell and dendritic cell activity and lesional epidermal hyperplasia, while increasing the expression of barrier and lipid metabolism genes, such as filaggrin [3]. Pharmacologic targeting of this immune dysregulation is associated with improvement in clinical disease severity [3].

Dupilumab is approved by the Food and Drug Administration (FDA) as a treatment for moderate-to-severe AD in patients aged six months and older [2]. This novel drug has demonstrated marked improvements in the severity of AD symptoms through multiple clinical trials. Despite these promising results, there is still a need to assess and monitor the safety of long-term use [2]. Dupilumab is tolerated well by most patients, but side effects such as injection site reactions, herpes simplex infection, temporary increases in eosinophil levels, and conjunctivitis have been reported [4]. Other reported dupilumab-associated ocular diseases include keratitis and blepharitis [5]. These effects have been collectively referred to as dupilumab-associated ocular surface disease (DAOSD) in the literature.   

Although conjunctivitis is a widely recognized side effect of the use of dupilumab, there are very few studies that describe patient variables associated with this adverse event. There are no standardized recommendations for monitoring patients on this medication. This study aims to highlight the risk factors associated with the development of DAOSD described in the current literature.

This article was previously presented as a meeting abstract at the 2024 Michigan Osteopathic Association Annual Spring Scientific Research Exhibit Competition and Awards on May 18, 2024, and at the 2024 Revolutionizing Alopecia Areata, Vitiligo, and Eczema Conference on June 8, 2024.

## Review

Methods

Framework

**Figure 1:**
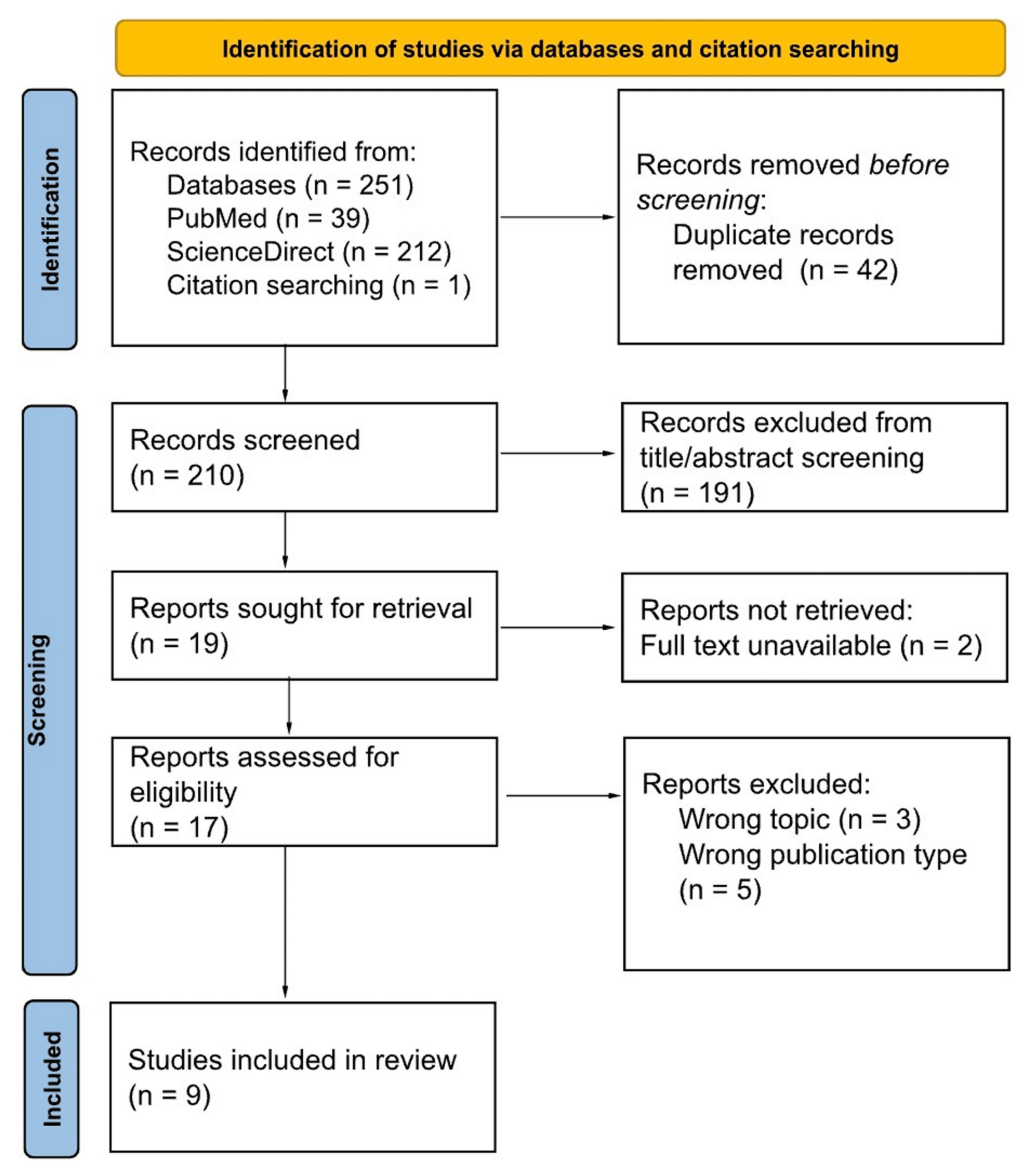
PRISMA flowchart outlining study identification and selection PRISMA: Preferred Reporting Items for Systematic Reviews and Meta-Analyses

This scoping review was guided by the structure described by Arksey and O’Malley [6], Peters et al. [7], and the Joanna Briggs Institute [8]. The process utilized the Preferred Items for Systematic Reviews and Meta-Analyses extension for Scoping Reviews (PRISMA-ScR) checklist [9]. The phases of this process include identifying studies, selecting those that meet set criteria, extracting data, and synthesizing the findings. The PRISMA flow diagram developed by Page et al. is included in Figure 1 and depicts the number of studies at each step of the identification and selection process.

Search Process

A search of the PubMed and ScienceDirect research databases was conducted by the primary reviewer in April 2024. Nonsystematic searches were first performed to determine commonly referenced terms in the literature regarding the topic. Search terms used to identify relevant studies included “dupilumab”, “atopic dermatitis”, “risk factors”, “demographics”, and various terms to represent ocular sequelae including “keratitis”, “keratoconjunctivitis”, and “conjunctivitis”. A combination of Boolean operators and the previously mentioned search terms was used to conduct systematic searches of the databases to identify papers that are relevant to the study objective. 

Inclusion and Exclusion Criteria 

Original peer-reviewed articles were included in this study with no criteria for date of publication, as dupilumab was approved for use in 2017 and studies have been published thereafter. Studies were included if they were published in English and included a discussion of risk factors for ocular side effects with dupilumab treatment. Studies were excluded if they discussed ocular conditions of other origins or biological agents other than dupilumab. In an effort to minimize redundancy, systematic reviews were excluded from the study. Other article types excluded were non-peer-reviewed studies and review papers.

*Study Selection* 

The searches of PubMed and ScienceDirect databases with the above search terms yielded a total of 251 papers, with one additional paper found through citation searching. A total of 210 articles were screened by all four reviewers after 42 duplicates were removed. Inconclusive decisions on article inclusion were reviewed by two authors to determine inclusion or exclusion. The 17 papers that met screening criteria and were accessible were retrieved for inclusion in a full-text review. Two reviewers conducted a full-text review to determine appropriateness for inclusion and nine papers remained for analysis.

Data Collection and Synthesis

Data extraction from the selected articles was conducted by all reviewers, utilizing a computerized spreadsheet to organize data independently gathered by each reviewer. All data extraction was assessed by one reviewer for accuracy by cross-checking recorded data with the article source. Data were organized into groups during data synthesis and reported in the Results section. Any variance or concerns were addressed with all four reviewers during the data synthesis and revision process. Analyses were conducted numerically and thematically using the extracted data, including cited risk factors, whether an ophthalmological evaluation was recommended, and whether patients discontinued treatment as a result of ocular side effects.

Results

Across all reviewed studies, a history of both self-reported and diagnosed ocular disease was the most frequently cited risk factor for DAOSD. In the prospective cohort study by Achten et al. [10], 152/469 patients developed DAOSD. Of those 152 patients, 37 reported a history of eye disease, including atopic keratoconjunctivitis, giant papillary conjunctivitis, or vernal keratoconjunctivitis [10]. This association was also noted by Touhouche et al. [11], whose study revealed an increased frequency of dupilumab-associated ocular adverse events in patients with pre-existing dry eye syndrome or keratitis at baseline. Conversely, Pradhan et al. [12] found that a history of allergic conjunctivitis was not associated with the presence of DAOSD or its severity.  

Severe AD was highlighted as a risk factor for DAOSD in five out of the nine studies [12-16]. In the retrospective cohort study conducted by Nahum et al. [13], 16 out of 37 patients experienced new or worsened ocular symptoms following a two-week trial of dupilumab treatment. Sixteen out of 16 patients had severe AD, based on the Investigator Global Assessment (IGA) severity score [13]. The IGA score ranges from zero to five, with four and five representing severe and very severe, respectively [13]. None of the 16 patients had moderate dermatitis based on this severity score [13]. Moreover, this study’s results reported that a family history of atopy was protective against DAOSD [13]. This is contrasted with the findings of the prospective observational study by Nettis et al. [14], in which a family history of atopy was noted to be a risk factor for DAOSD. In addition, DAOSD was reported to be intolerable for some patients and subsequently caused them to discontinue treatment. In a study by Treister et al. [15], three patients temporarily or permanently discontinued treatment due to ocular side effects, and all three patients were noted to have severe AD or at least one other atopic condition.

Four out of nine studies described elevated total immunoglobulin E (IgE) levels and eosinophil count as risk factors for DAOSD. Of note, one study described the relationship between high levels of chemokines and DAOSD. Results from Uchida et al. [17] revealed that patients who developed conjunctivitis after starting dupilumab had higher baseline serum levels of thymus and activation-regulated chemokine (TARC) and IgE than patients who did not. However, no clinical significance in severity was noted among the patients with higher TARC and IgE levels [17]. Barbé et al. [16] conducted a retrospective cohort study, which revealed transient eosinophilia at baseline in 12 out of 18 patients who developed DAOSD.

Facial and/or eyelid eczema was described as a notable risk factor for DAOSD in three out of the nine studies. In the prospective study by Costedoat et al. [18], head and neck AD at baseline was found to be an independent risk factor for dupilumab-induced blepharoconjunctivitis. Erythroderma and the presence of dry eye syndrome at baseline were also noted to be independent risk factors in this study [18]. 

Discussion 

In this review, nine articles were identified that addressed the manifestation of ocular side effects in patients with AD being treated with dupilumab, commonly referred to as DAOSD. The main ocular side effects that comprise DAOSD include conjunctivitis, keratitis, and dry eye syndrome. Upon review of the literature, the connection between AD treated with dupilumab and ocular side effects remains largely undetermined in the scientific community. Some studies suggest that having a higher baseline level of TARC and IgE may be associated with DAOSD [10-11,14,17], whereas others correlate the ocular sequelae to the severity of AD [12-16] or having a history of ocular disease prior to dupilumab initiation [10-11,13-14,17-18].

In past studies, the severity of AD has been implicated in the development of DAOSD. Treister et al. [15] noted that nine out of 12 patients who developed ocular side effects scored a 4 on the IGA scale, indicating widespread, severe atopic disease. Similarly, a study by Barbé et al. [16] found that 72% of the patients who developed ocular side effects had a more severe presentation of AD before starting treatment with dupilumab, determined by a SCORAD score above 50. However, ocular side effects were not seen as frequently with other atopic diseases, such as allergic rhinitis and allergic conjunctivitis, indicating that there is likely some predisposition to DAOSD in patients who have severe AD [19].

Six out of nine articles touched on a history of ocular disease as an independent risk factor for DAOSD. In the study by Uchida et al. [17], every patient with a prior history of AKC developed DAOSD, implying a likely association between the two. However, because some studies did not require a baseline ocular screening before beginning their study, it is unknown if previous ocular disease is a definitive risk factor for developing DAOSD. In order to determine whether previous ocular history plays a role in developing ocular side effects with dupilumab, it is necessary for ophthalmologists to document ocular disease and extensively evaluate patients before the initiation of treatment.

Ophthalmologic Evaluation Prior to/During Treatment

Although the relationship between severe AD treated with dupilumab and ocular disease is yet to be defined, data from the included studies indicate the necessity of ophthalmologic evaluation before treatment initiation. Per the initial literature search, there are no standardized guidelines regarding the management of DAOSD. Out of the nine studies evaluated, six indicated that baseline ocular disease potentially played a role in or increased the risk of developing DAOSD [10-11,13-14,17-18]. Based on these results, we recommend encouraging patients at high risk of developing DAOSD to be screened by an ophthalmologist before the initiation of dupilumab. Both dermatologic and ophthalmologic evaluations may help to determine an individual's candidacy for dupilumab and their risk for potential adverse ocular effects. ​​​

Need to Discontinue Treatment Due to Adverse Effects

Data from Akinlade et al. [20] revealed that those with a more severe form of AD were more likely to experience DAOSD. Consequently, many of these patients discontinued dupilumab use due to the development of severe conjunctivitis despite a favorable dermatologic response to treatment [20]. Another study from Treister et al. [15] described two patients with a history of hay fever who discontinued dupilumab, further suggesting that a history of atopic disease may be closely related to the development of DAOSD as well. Achten et al. [21] found that increasing the dose interval between dupilumab doses from 300 mg every three weeks to 300 mg every five weeks improved symptoms of DAOSD. By altering the dosing frequency and/or quantity of dupilumab, it may be possible to continue the drug in patients who are exhibiting a positive treatment response without incurring the morbidity of ocular side effects. Although this study took place in an AD expertise center where all patients exhibited moderate-to-severe AD, its results may provide guidance for all clinicians managing patients with DAOSD.

Management of DAOSD When It Develops

The purpose of this paper was to review risk factors for DAOSD, and therefore, an extensive review of its proposed management will not be reviewed here. It is generally recommended to treat DAOSD with lubricating eye drops, as well as anti-inflammatory therapies including topical ophthalmic steroids and/or immunomodulators, such as tacrolimus skin ointment [22].  

*Limitations of the Study * 

Upon review of the selected articles, there were some limitations noted. Only one out of the nine articles was based in the United States, and some studies were performed in settings that do not accurately represent patients who are seen on a daily basis (e.g. an AD expertise center). In addition, ophthalmologic examination prior to dupilumab initiation was not a prerequisite for some of these studies. This may lessen the significance of the results as it is not known if ocular disease was present prior to initiation of dupilumab treatment [22].

*Future Work * 

Further studies investigating DAOSD are necessary to make informed decisions about an individualized management plan for each patient. Studies may investigate whether a history of any atopic disease definitively places patients at higher risk of DAOSD, when clinicians can expect to encounter ocular side effects in patients receiving dupilumab therapy, and whether different severities of AD influence the development of DAOSD. Distinguishing the pathophysiology of ocular disease development with the use of dupilumab may help clinicians fully understand the risks patients incur when starting treatment. In addition, prospective studies that recruit patients from a clinic, rather than retrospective studies of clinical trials, may better allow results to be extrapolated to the entire population of individuals with AD and represent how dupilumab affects patients and their quality of life. 

## Conclusions

Multiple risk factors for the development of DAOSD were identified in this review. A history of ocular disease and a more severe phenotype of atopic dermatitis were among the most commonly cited risk factors for DAOSD. The ocular side effects from treatment may cause patients to discontinue dupilumab treatment despite a positive dermatologic response to treatment, ultimately affecting their quality of life. Recognition of and screening for these risk factors early on by ophthalmologists and dermatologists may have a beneficial role in both the management of atopic dermatitis and the management of ocular side effects. Additional studies are needed to explore these associations and their implications for patient care.

## References

[1] Langan SM, Irvine AD, Weidinger S (2020). Atopic dermatitis. Lancet.

[2] Gooderham MJ, Hong HC, Eshtiaghi P, Papp KA (2018). Dupilumab: a review of its use in the treatment of atopic dermatitis. J Am Acad Dermatol.

[3] Guttman-Yassky E, Bissonnette R, Ungar B (2019). Dupilumab progressively improves systemic and cutaneous abnormalities in patients with atopic dermatitis. J Allergy Clin Immunol.

[4] Harb H, Chatila TA (2020). Mechanisms of dupilumab. Clin Exp Allergy.

[5] Wu D, Daniel BS, Lai AJ (2022). Dupilumab-associated ocular manifestations: a review of clinical presentations and management. Surv Ophthalmol.

[6] Arksey H, O'Malley L (2005). Scoping studies: towards a methodological framework. Int J Soc Res Methodol.

[7] Peters MD, Marnie C, Tricco AC (2020). Updated methodological guidance for the conduct of scoping reviews. JBI Evid Synth.

[8] Peters MD, Godfrey C, McInerney P, Munn Z, Tricco AC, Khalil H (2024). Scoping reviews. JBI Manual for Evidence Synthesis.

[9] Page MJ, McKenzie JE, Bossuyt PM (2021). The PRISMA 2020 statement: an updated guideline for reporting systematic reviews. BMJ.

[10] Achten RE, Van Luijk C, Van der Rijst L (2022). Identification of risk factors for dupilumab-associated ocular surface disease in patients with atopic dermatitis. Acta Derm Venereol.

[11] Touhouche AT, Cassagne M, Bérard E (2021). Incidence and risk factors for dupilumab associated ocular adverse events: a real-life prospective study. J Eur Acad Dermatol Venereol.

[12] Pradhan SP, Sadiq SN, Cartes C (2024). Dupilumab induced ocular surface disease: a prospective case series. Eur J Ophthalmol.

[13] Nahum Y, Mimouni M, Livny E, Bahar I, Hodak E, Leshem YA (2020). Dupilumab-induced ocular surface disease (DIOSD) in patients with atopic dermatitis: clinical presentation, risk factors for development and outcomes of treatment with tacrolimus ointment. Br J Ophthalmol.

[14] Nettis E, Bonzano L, Patella V, Detoraki A, Trerotoli P, Lombardo C (2020). Dupilumab-associated conjunctivitis in patients with atopic dermatitis: a multicenter real-life experience. J Investig Allergol Clin Immunol.

[15] Treister AD, Kraff-Cooper C, Lio PA (2018). Risk factors for dupilumab-associated conjunctivitis in patients with atopic dermatitis. JAMA Dermatol.

[16] Barbé J, Poreaux C, Remen T (2021). Prevalence of ocular disease during dupilumab treatment for atopic dermatitis: a bicentric retrospective comparative cohort study. Int J Dermatol.

[17] Uchida H, Kamata M, Nagata M (2020). Conjunctivitis in patients with atopic dermatitis treated with dupilumab is associated with higher baseline serum levels of immunoglobulin E and thymus and activation-regulated chemokine but not clinical severity in a real-world setting. J Am Acad Dermatol.

[18] Costedoat I, Wallaert M, Gaultier A (2023). Multicenter prospective observational study of dupilumab-induced ocular events in atopic dermatitis patients. J Eur Acad Dermatol Venereol.

[19] Maudinet A, Law-Koune S, Duretz C, Lasek A, Modiano P, Tran TH (2019). Ocular surface diseases induced by dupilumab in severe atopic dermatitis. Ophthalmol Ther.

[20] Akinlade B, Guttman-Yassky E, de Bruin-Weller M (2019). Conjunctivitis in dupilumab clinical trials. Br J Dermatol.

[21] Achten R, Bakker D, Ariens L (2021). Long-term follow-up and treatment outcomes of conjunctivitis during dupilumab treatment in patients with moderate-to-severe atopic dermatitis. J Allergy Clin Immunol Pract.

[22] Achten R, Thijs J, van der Wal M (2024). Ocular surface disease in moderate-to-severe atopic dermatitis patients and the effect of biological therapy. Clin Exp Allergy.

